# Back to the future: evolving bacteriophages to increase their effectiveness against the pathogen *Pseudomonas aeruginosa* PAO1

**DOI:** 10.1111/eva.12085

**Published:** 2013-07-15

**Authors:** Alex Betts, Marie Vasse, Oliver Kaltz, Michael E Hochberg

**Affiliations:** 1 Institut des Sciences de l'Evolution, UMR 5554, Université Montpellier 2Montpellier CEDEX 05, France; 2 Santa Fe InstituteSanta Fe, NM, USA

**Keywords:** coevolution, cross-infection, cystic fibrosis, disinfection, evolutionary engineering, experimental evolution, nosocomial, phage therapy, phage training, resistance

## Abstract

Antibiotic resistance is becoming increasingly problematic for the treatment of infectious disease in both humans and livestock. The bacterium *Pseudomonas aeruginosa* is often found to be resistant to multiple antibiotics and causes high patient mortality in hospitals. Bacteriophages represent a potential option to combat pathogenic bacteria through their application in phage therapy. Here, we capitalize on previous studies showing how evolution may increase phage infection capacity relative to ancestral genotypes. We passaged four different phage isolates (podoviridae, myoviridae) through six serial transfers on the ancestral strain of *Pseudomonas aeruginosa* PAO1. We first demonstrate that repeated serial passage on ancestral bacteria increases infection capacity of bacteriophage on ancestral hosts and on those evolved for one transfer. This result is confirmed when examining the ability of evolved phage to reduce ancestral host population sizes. Second, through interaction with a single bacteriophage for 24 h, *P. aeruginosa* can evolve resistance to the ancestor of that bacteriophage; this also provides these evolved bacteria with cross-resistance to the other three bacteriophages. We discuss how the evolutionary training of phages could be employed as effective means of combatting bacterial infections or disinfecting surfaces in hospital settings, with reduced risk of bacterial resistance compared with conventional methods.

## Introduction

Antibiotics are still the most potent weapon to treat bacterial infections, but the evolution of resistance is becoming increasingly problematic both in hospitals (Kutateladze and Adamia 2010) and in agriculture (Johnston 2002). Resistance genes predate the therapeutic use of antibiotics and have rapidly increased in frequency in response to antibiotic use (D'Costa et al. 2011). Consequently, there is mounting interest in the search for alternatives to antibiotics.

One such alternative is the therapeutic use of bacteriophages (e.g. Kutateladze and Adamia 2010; Loc-Carrillo and Abedon 2011; Pirnay et al. 2012). In fact, bacteriophages were used to treat bacterial infections prior to the discovery and medical use of antibiotics (Lu and Koeris 2011). However, antibiotics gained popularity in the Western world due to advantages such as their broad spectrum and ease of production, despite phage therapy being a viable alternative (Summers 2001). Phage therapy possesses several advantages over antibiotics (Loc-Carrillo and Abedon 2011). For example, the narrow host ranges of many bacteriophages allow their employment as therapeutics without affecting the natural flora of patients (Kutateladze and Adamia 2010), and unlike antibiotics, some can evolve to counter the adaptations of resistant bacteria (Debarbieux et al. 2010). Additionally, lytic phages may actually increase in concentration at the site of infection (Carlton 1999) without causing added toxicity (Summers 2001). Bacteriophages could also have potential applications as disinfectants (VinodKumar et al. 2010), which could help prevent infections in patients, potentially reducing antibiotic use.

Here, we consider *Pseudomonas aeruginosa* as a potential target of phage therapy. This Gram-negative bacterium is one of the most common causes of nosocomial infections (Driscoll et al. 2007) and represents the second most likely source of hospital-acquired bacterial pneumonia (Jones 2010), associated with particularly high mortality in patients requiring mechanical breathing assistance (Chastre and Fagon 2002). It is also responsible for debilitating, chronic lung infections, resulting in high mortality for patients with cystic fibrosis (Driscoll et al. 2007), causes postsurgical complications (Secher et al. 2005) and infects wounds or burns, increasing patient mortality (Babu et al. 2011). Outbreaks can also be devastating for infants (RQIA 2012). Moreover, this pathogen is extremely versatile and can colonize a large range of environmental niches including food and drinking water (Hardalo and Edberg 1997).

Described as ‘a phenomenon of bacterial resistance’ (Strateva and Yordanov 2009), *P. aeruginosa* strains isolated from hospitals have been found to resist as many as 16 different antibiotics (Deredjian et al. 2011), and multidrug-resistant *P. aeruginosa* infections are becoming increasingly common (Gomes et al. 2011). Recent experiments have investigated the potential of phage therapy to combat *P. aeruginosa*. *In vitro* experiments have demonstrated the capacity of phage to reduce bacterial population size (Hall et al. 2012), and even dissolve the defensive biofilms of *P. aeruginosa* (Harper and Enright 2011; Pires et al. 2011), which may help increase the efficacy of existing antibiotics (Verma et al. 2010). *In vivo* studies showed that phages also reduce or even prevent infection in insect and mouse model systems (Khawaldeh et al. 2011; Morello et al. 2011).

However, there are a number of recognized challenges to phage therapy, including insufficient numbers of phages introduced, the prior existence or evolution of bacterial resistance, and bacterial inaccessibility or protective structures such as biofilms (for discussions see, e.g. Abedon 2011, 2012; Kutter et al. 2009; Harcombe and Bull 2005; Levin and Bull 2004; Scott et al. 2007). Nevertheless, there are a number of ways to improve phage effectiveness based on ecological and evolutionary approaches, including selection for increased adsorption (Uchiyama et al. 2011), combination therapies with antibiotics (Lu and Collins 2009), the use of phage cocktails (Carvalho et al. 2010; Chan and Abedon 2012) and selection on optimal mutation rates (Kysela and Turner 2007). Moreover, bacteriophages are well known for their capacity to rapidly adapt to bacterial host populations (e.g. Buckling and Rainey 2002; Buckling et al. 2009). This strong evolutionary potential may help select phages that are specifically designed to attack wider ranges of *P. aeruginosa* genotypes within an infection than would (ancestral) phages sampled from the environment. A simple way of engineering therapeutic phage consists of ‘training’ (Pirnay et al. 2012), for example adapting phage to target bacteria *in vitro* or *in vivo*. This can be performed by way of serial passage, a form of experimental evolution that is used, for example in vaccine development (Ebert 1998). Such experiments involve the continuous culture of parasites by supplying them always with the same (naive) clonal host population at each passage (Ebert 1998). As a result, passaged parasites usually become increasingly effective at attacking the host on which they have been passaged (Ebert 1998; Poullain et al. 2008). Hence, phage therapy strategies may use this technique to evolve the phage ‘into the future’ by means of serial passage, and then confront the improved phage with its bacterial past in an actual infection. Because of the short generation times, large population sizes and the ease of culturing bacteria and phage, this approach may allow the targeting and selection of engineered phage in real time, within the time span of a particular infection (Pirnay et al. 2012). Morello et al. (2011) report a case of rapid increase in phage efficacy against a clinical *P. aeruginosa* strain after only five cycles of serial passage. However, not all phage isolates are equally efficient, and some studies also report resistance evolution in long-term cultures (Hall et al. 2012; Pires et al. 2011), suggesting the possibility of coevolutionary arms races between phage and bacteria. This may limit the success of phages both during the selection phase and during the treatment of infection.

We evaluated several factors that are likely to be important in using serial passage for the improvement of phage infection capacity on pathogenic bacteria. First, we examined to what extent passaged bacteriophage isolates (hereafter called ‘evolved phage’ or ‘trained phage’) gained in infection capacity when confronted with target bacteria (i.e. ancestors). Second, assuming that one of the goals of serial passaging is to overcome bacterial resistance to natural (unevolved) phage, we confronted evolved bacteria both with trained phage and with familiar and unfamiliar isolates of natural phage. We report for the first time to our knowledge evidence that *P. aeruginosa* has the potential to coevolve with different lytic phage isolates. Repeated serial passage on ancestral bacteria increased bacteriophage infection capacity. Conversely, bacteria evolved resistance to bacteriophage ancestors and cross-resistance to the other bacteriophages during a single passage episode. We argue that the idea that ‘evolutionary training’ is a powerful tool to produce more effective phages for use in eliminating or controlling nosocomial bacterial infections or disinfecting surfaces in hospital settings.

## Materials and methods

### Isolating bacterial colonies and culture growth conditions

Single colonies of *P. aeruginosa* PAO1 were isolated from an exponentially growing stock population by plating a diluted sample onto a KB agar plate (12 g/L agar) and by incubating it at 37°C for 24 h.

Bacteria were cultured in 30-mL plastic Falcon tubes (microcosms) containing 6 mL King's B (KB) media (King et al. 1954). Each microcosm was inoculated with a single colony of ancestral PAO1 then incubated at 37°C and 200 rpm continuous orbital agitation for 24 h.

### Ancestral bacteriophage stocks

We obtained four phage isolates previously investigated for their infectivity on *P. aeruginosa,* three from the podoviridae (*LKD16*, *PEV2* and *LUZ7*) and one from the myoviridae (*14/1*) (Ceyssens et al. 2006, 2009, 2010). Samples of these stocks were stored at 4°C as purified supernatants in liquid KB. Each phage isolate was amplified from one arbitrarily chosen plaque and was introduced into a single exponentially growing PAO1 culture. After 24 h, 1 mL aliquots of each phage-containing culture was then added to 110 μL chloroform to kill bacteria and then vortexed for 10 seconds. The samples were then centrifuged at 13 000 rpm for 4 min. Finally, the phage-containing supernatants were each carefully recovered and pipetted into 1-mL Eppendorf tubes and stored at 4°C. These four stocks (each *c*. 1 × 10^7^ PFU/mL) were then used as the ancestral phages for these experiments.

### Establishing replicate populations

Each replicate consisted of a 6-mL KB microcosm inoculated with 60 μL of an overnight ancestral PAO1 culture (‘*t*_0_’) and a sample of a single arbitrarily chosen phage stock. Eight replicate populations were established for each of the four ancestral phage isolates. The microcosms were then incubated 37°C at 200 rpm.

### Serial passage experiment

**Figure 1:**
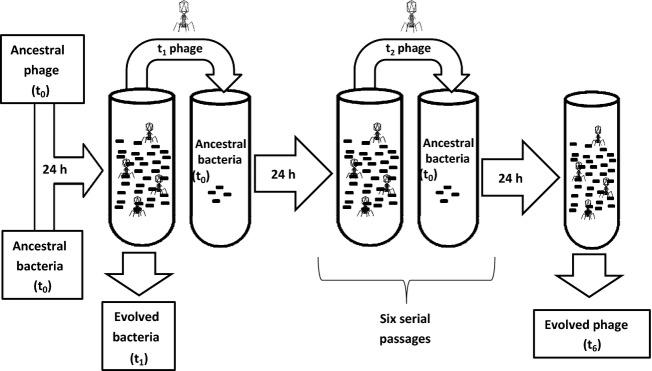
Design of the serial passage experiment. Phage were allowed to amplify on naive (*t*_0_) bacteria (strain PAO1 of *Pseudomonas aeruginosa*) for 24 h, then bacteria and phage were separated. The recovered *t*_1_ phage population was then transferred to a new tube containing naive *t*_0_ bacteria. After another 24 h of amplification, bacteria and phage were again separated and the recovered *t*_2_ phage population amplified on *t*_0_ bacteria, and so on. Thus, six serial passages were accomplished, for four different phage isolates (*PEV2*, *LUZ7*, *LKD16*, *14/1*). For tests of evolutionary change in infection capacity, we used evolved phage (*t*_6_) and ancestral phage (*t*_0_). These phage types were tested against the naive *t*_0_ bacteria as well as *t*_1_ bacteria, recovered after the first round of exposure to phage. The bacterial response to phage-mediated selection was assessed by comparing resistance between *t*_0_ and *t*_1_ bacteria.

Phages were passaged by incubating them for 24 h under the conditions described above, vortexing and then transferring 60 μL into an exponentially growing overnight PAO1 *t*_0_ culture. After 24 h of interaction with phage, ‘*t*_1_’ bacteria from each microcosm were isolated by vortexing and then by taking a 1-mL aliquot, which was then centrifuged at 13 000 rpm for 4 min. The supernatant was discarded, and the pellet was resuspended in 1 mL KB by vortexing. Washing was repeated three times to ensure any phages that may have remained were at very low numbers. The samples were then plated to obtain single colonies. After 6 serial passages, the resultant evolved phages (*t*_6_) were purified and stored at 4°C. The experimental design is summarized in Fig. 1.

### Measuring infection capacity

Infection capacity is defined here as the probability that an arbitrarily sampled bacterium is infected and killed by the phage. A 30-μL droplet of a vortexed bacteriophage population was applied to a square KB agar plate inclined slightly such that the droplet would run down the plate in a straight line. A sample of each of 20 bacterial colonies, taken arbitrarily with a sterile loop, was then streaked once through each phage line (while still liquid). The plates were then incubated for 24 h at 37°C, after which a bacterial colony was considered resistant to a given bacteriophage if its growth continued uninterrupted by the line of phage (Fig. S1).

At the end of experiment 1, infection capacity was measured for ancestral *t*_0_ phages (4 phage isolates × 8 replicates) and for evolved *t*_6_ phages (4 isolates × 8 replicate lines). Phages were confronted with the naive *t*_0_ bacteria and with *t*_1_ bacteria, with which they had interacted during the first passage cycle (total number of replicates in assay: 4 phage isolates × 8 replicates × 2 bacteria types = 64).

In addition to confrontation with their own *t*_0_ phage, *t*_1_ bacteria were also tested against the three foreign *t*_0_ phage isolates, with which they had not interacted. For this test, we arbitrarily chose two of the 8 populations of *t*_1_ bacteria interacting with a given phage isolate (4 bacterial *t*_1_ origins × 2 replicate lines × 3 foreign assay *t*_0_ phage isolates = 24 replicates). We were thus able to analyse a complete cross-infection matrix, with each of the four *t*_1_ bacterial origins being tested against each of the four *t*_0_ phage isolates (total = 56 replicates; Fig. S2).

### Measuring phage impact on bacterial population density

We assessed the impact of ancestral *t*_0_ phages compared with evolved *t*_6_ phages on the ancestral PAO1 bacterium. Bacterial populations were grown in 30-mL Falcon tubes containing 6 mL of KB at 37°C and 200 rpm continuous orbital agitation. During the bacterial exponential growth phase (6 h after inoculation in KB), replicates of bacterial cultures received *c*. 10^5^ phages from each of the four ancestral phage isolates (8 replicates per isolate) and from each evolved *t*_6_ phage (4 isolates × 8 evolved replicate lines). We also established 8 control replicates without adding phage. The assay cultures were grown for 18 additional hours under the same conditions as the main experiment. Bacterial CFUs were counted on KB agar plates at the beginning and at the end of the assay to estimate population densities.

### Statistical analysis

Phage infection capacity was analysed by means of logistic regression, which uses successful/unsuccessful attack of a bacterial colony as a binary response variable. In a factorial model, we tested effects of phage origin (4 isolates), phage type (ancestral *t*_0_, passaged *t*_6_) and bacterial type (naive *t*_0_, evolved *t*_1_) in experiment 1.

For the cross-infection assay, we also used logistic regression to analyse effects of bacterial *t*_1_ origin (evolved with one of the four phage isolates) and phage isolate (4 *t*_0_ isolates tested in the assay) on bacterial resistance (attack ‘yes/no’). We further tested for isolate-specific adaptation of bacteria by comparing sympatric (*t*_1_ bacteria against ‘own’ phage isolate) and allopatric (*t*_1_ bacteria against ‘foreign’ phage isolate) combinations of bacteria and phage. This was done by partitioning the variation explained by bacterial *t*_1_ origin × phage *t*_0_ isolate interaction to estimate the fraction of the variance explained by the difference between ‘own’ vs. ‘foreign’ pairings of bacteria and phage. In both analyses, selection line identity was considered as random factor.

For analysis of the capacity of the phage to reduce bacterial density, we used log-transformed estimates of bacterial density after 24 h of exposure to phage as the response variable, and phage origin and phage type as explanatory factors. Analyses were conducted with JMP V10 (SAS 2012) and SAS (SAS 1996) statistical packages.

## Results

### Evolution of phage infection capacity during serial passage

**Figure 2:**
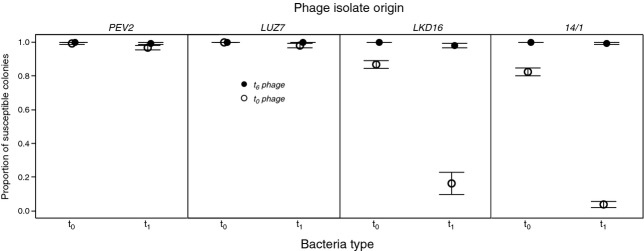
Mean (± SE) infection capacity of passage phage (*t*_6_) and ancestral phage (*t*_0_), measured on naive (*t*_0_) and evolved (*t*_1_) bacteria and shown for four genetic backgrounds of phage (isolates *PEV2*, *LUZ7*, *LKD16* and *14/1*). Phages were passaged for 6 cycles; evolved bacteria (*t*_1_) originate from a single *P. aeruginosa* strain (PAO1) and were isolated after the first cycle of serial passage of a given phage isolate.

In experiment 1, all passaged *t*_6_ phage selection lines showed a near 100% infection capacity (99.6 ± 0.002%) on both naive *t*_0_ bacteria and evolved *t*_1_ bacteria (Fig. 2). Statistical analysis showed that responses to selection in the phage varied with isolate identity (phage type × phage isolate origin interaction: *F*_3,28_ = 22.1, *P* < 0.0001; Table S1). Phage isolates *LKD16* and *14/1* initially infected 80–85% of the naive (*t*_0_) bacteria, and serial passage increased their infection capacity to nearly 100% (two right panels in Fig. 2). In contrast, infection capacity of ancestral phage isolates *PEV2* and *LUZ7* was already near 100%, and the selection lines simply retained this high level during serial passage (two left panels in Fig. 2).

### Evolution of bacterial resistance during the first cycle of serial passage

Bacterial evolution was inferred from comparison of resistance between naive *t*_0_ bacteria and *t*_1_ bacteria, exposed to phage during the first passage cycle. As for phage evolution, bacterial evolution depended on phage isolate identity (bacteria type × phage isolate origin interaction: *F*_3,28_ = 4.5, *P* = 0.0328; Table S1). There was a substantial increase in bacterial resistance against phages *LKD16* and *14/1*, reducing phage infection capacity from initially 80–85% to less than 20% (Fig. 2). In contrast, we observed only a marginal increase in resistance against the two more infectious phages (*PEV2* and *LUZ7*), with the majority of *t*_1_ bacteria (>95%) still being susceptible to these two phage isolates (Fig. 2).

**Figure 3:**
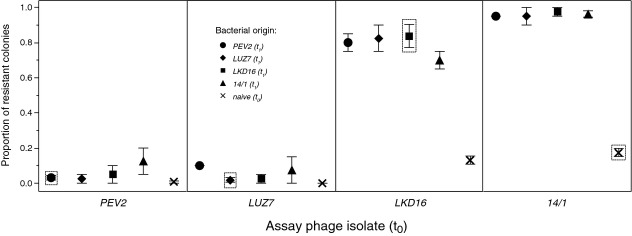
Mean (± SE) resistance of evolved (*t*_1_) bacteria against four phage isolates. Bacterial origin denotes the phage isolate (*PEV2*, *LUZ7*, *LKD16* or *14/1*), to which bacteria were exposed during the first of 6 cycles of serial passage of the phage. The bacteria were tested against the ancestral, nonpassaged, phage isolates (*t*_0_). To illustrate the response to phage-mediated selection for resistance in the *t*_1_ bacteria we show the level of resistance of the naive (*t*_0_) bacteria to each of the four phage isolates. Rectangles indicate the resistance of *t*_1_ bacteria to their ‘own’ phage, with which they evolved for 24 h. All other estimates represent levels of cross-resistance to ‘foreign’ phage, to which *t*_1_ bacteria had no prior exposure.

The cross-infection experiment revealed a similar picture of high resistance of *t*_1_ bacteria to phages *PEV2* and *LUZ7* (>80%) and low resistance to *LKD16* and *14/1* (≍ 15%; Fig. 3; effect of assay phage isolate: *F*_3,12_ = 377, *P* < 0.0001). Statistical analysis showed a significant phage isolate × bacteria origin interaction (*F*_9,28_ = 6.1, *P* = 0.0025; Table S2), but there was no evidence for specific adaptation of the *t*_1_ bacteria to their own phage (comparison of ‘own’ vs. ‘foreign’ pairings of *t*_1_ bacteria and *t*_0_ phage: *F*_1,8_ ≪ 1, n.s.).

Most bacteria evolved some level of cross-resistance to ‘foreign’ phage, to which they had no prior exposure. This can be seen in Figure 3, where *t*_1_ bacteria generally were more resistant to foreign *t*_0_ phages than the naive *t*_0_ bacteria (*t*_23_ = 6.43, *P* < 0.0001). The level of evolved cross-resistance varied substantially with identity of the phage isolate, on which resistance was assayed. However, cross-resistance could not be predicted from the evolutionary past of one bacterium. For example, a large gain in resistance to own phage *LKD16* was associated with a strong cross-resistance to one phage (*14/1*) and with weak cross-resistance to the two other phages (*PEV2* and *LUZ7*; Fig. 3). Indeed, overall, there was no significant correlation between direct response to selection (increase in resistance to own phage) and the correlated response to selection (increase in resistance to foreign phage) (*r* = −0.24, *n* = 24, *P* = 0.2503, across all bacterial replicate lines combined).

### Phage impact on bacterial population density

**Figure 4:**
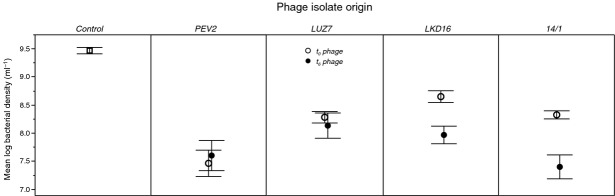
Mean (± SE) bacterial density after 24 h of exposure to ancestral *t*_0_ and evolved *t*_6_ phages, shown for four phage isolate origins (*PEV2*, *LUZ7*, *LKD16* and *14/1*). Phages were tested on naive *t*_0_ bacteria. In the control treatment, bacteria were not exposed to phage.

Phage reduced bacterial population density by up to 2 orders of magnitude (control vs. phage: *t*_62_ = 6.68, *P* < 0.0001; Fig. 4). The degree of this density reduction depended on both phage isolate and type (significant interaction between the two factors: *F*_3,48_ = 3.76, *P* = 0.0166; Table S3). Thus, the *t*_6_ phages derived from isolates *LKD16* and *14/1* were significantly better in reducing density than their *t*_0_ ancestral phages; in contrast, there was no significant difference between *t*_6_ and *t*_0_ phages for *PEV2* and *LUZ7* phage origins (Fig. 4). These differential evolutionary responses mirror those for infection capacity, where an increase was observed for the former two isolates, but not for the latter two (Fig. 2). Similarly, as for infection capacity, the ancestral *t*_0_ isolates differed significantly in the capacity to reduce bacterial density (*F*_3,27_ = 13.28, *P* < 0.0001), whereas evolved *t*_6_ phages were not significantly different with respect to their isolate origin (*F*_3,28_ = 0.48, *P* > 0.7).

## Discussion

Our results show that passaged phage increases both the range of bacterial clones infected and killed and the impact on bacterial populations, such that by the end of the experiment, all four phages had evolved to the point where the original PAO1 population was virtually 100% susceptible to infection. Even when the bacteria were evolved in the presence of phage for one transfer, colonies resistant to the trained phage were still at low frequency within the population. These results are consistent with basic predictions of how passaging parasites and pathogens can increase their host ranges and impacts on host populations (Ebert 1998; Poullain et al. 2008), and numerous experimental evolution studies (Buckling and Rainey 2002; Buckling et al. 2006; Poullain et al. 2008; Gaba and Ebert 2009) indicating that trait evolution is predictable and can be controlled to some extent in the laboratory. Our finding that phage can be rapidly evolved to largely overcome the initial steps of bacterial resistance clearly highlights the potential of this approach for increasing the effectiveness of phage therapy and disinfection of hospital environments.

We report what is to our knowledge the first evidence of bacteria–phage coevolution involving the bacterium *P. aeruginosa*, although we only investigated this phenomenon for a single serial transfer. Previous study (Brockhurst et al. 2005; Hall et al. 2012) showed evolution of resistance in *P. aeruginosa*, but did not examine phage evolution. We found that evolving phages with bacteria increased the former's host range against *t*_0_ bacteria and that *t*_1_ bacteria had enhanced resistance against *t*_0_ phage, indicative of a coevolutionary process (e.g. Gaba and Ebert 2009; but see Nuismer et al. 2010). However, we cannot exclude the possibility that the enhanced infectivity of *t*_6_ phage on both *t*_0_ and *t*_1_ bacteria was only acquired during the first 24 h of interaction between the bacteria and phage. Our assays did not address the possibility that any (co)evolution occurring persisted beyond the first transfer. We therefore urge caution in the interpretation of our results; further investigation employing fully factorial time-shift experiments (Blanquart and Gandon 2013) is necessary to evaluate our claim.

Although our results are indicative of reciprocal adaptations following selection on traits influencing relative fitness, it is not known at present what traits may be involved. Previous study has however reported costs of resistance in *P. aeruginosa* PAO1 against various phages, including *14/1* (Brockhurst et al. 2005; Hall et al. 2012). Recent study suggests that coevolutionary interactions between bacteria and their lytic phage involve adaptations to the initial phases of the interaction, that is, tail fibres in the phage (Paterson et al. 2010) and surface proteins in the bacterium (e.g. Chapman-McQuiston and Wu 2008). However, evidence is also emerging that intracellular mechanisms may constitute selective barriers to phage proliferation and thus cell mortality (e.g. Garneau et al. 2010; Vale and Little 2010) and that bacteria may limit phage damage through plastic life-history changes (Poisot et al. 2012). Although there is evidence for different forms of selection with time elapsed in coevolutionary interactions (Hall et al. 2011), whether there is more than one trait involved and what multiple traits may be, remain unknown.

In many systems, interactions between hosts and parasites involve the evolution of specificity, both within and between species (e.g. Lohse et al. 2006; Sicard et al. 2007; Poisot et al. 2011a; Koskella et al. 2012). Our cross-infection assay revealed no evidence for specificity in resistance evolution among the *t*_1_ bacteria. Indeed, there was no difference between the gain in resistance against own and foreign phage isolates; variation in the response to selection mainly depended on the identity of the phage isolate, on which the evolved *t*_1_ bacteria were assayed. This suggests that cross-resistance might easily evolve in *P. aeruginosa*, and even be stably maintained over multiple generations, as indicated by Hall et al. (2012). Although evidence for coevolution in our system is preliminary, our findings are also consistent with arms-race type coevolutionary dynamics between *P. fluorescens* and its phage, where increases in bacterial resistance are relatively unspecific and involve substantial levels of cross-resistance (Brockhurst et al. 2007). Similar to multidrug resistance in antibiotic therapies (Li and Nikaido 2009; Toprak et al. 2012), cross-resistance may be problematic for phage therapy. Nonetheless, we show that even relatively strong resistance can be overcome by the training of phage through serial passage.

Although all four phages tested successfully attacked a range of *t*_0_ bacteria, their host ranges differed considerably. *PEV2* and *LUZ7* were not significantly different from one another, but were significantly more virulent than *LKD16* and *14/1*. After experimental evolution, the four trained phages had all evolved >95% infection capacity to the ancestral bacterium (i.e. all 20 bacterial colonies tested were affected). Based on quantification of actual population reduction in *t*_0_ bacteria submitted to trained phage (*t*_6_), we suggest that our measure of infection capacity could be used in clinical situations to predict population impact. Indeed, previous study on *P. fluorescens* SBW25 and its lytic phage PHI2 suggests that coevolution increases the range of host genotypes exploited, whereas passaging increases the population level impact on the ancestral bacterium (Poullain et al. 2008), and this latter result is consistent with our observations on *P. aeruginosa* PAO1. We were unable to determine whether specific phage genotypes generated through the initial 24 h of evolution were lost in subsequent passaging, meaning that an improved method for assembling a trained phage sample in the context of treatments would be to include phage isolates from each round of passaging. Indeed, Hall et al. (2012) showed that multiple phage treatment had a stronger impact on bacterial population size than treatment with single phage isolates. Nevertheless, population level impacts of trained phage on *P. aeruginosa* PAO1 remain an open question, which is important to understand in deciding whether evolutionary training can improve phages for combatting bacterial pathogens.

Our methodological approach is an oversimplification of how natural *P. aeruginosa* populations could be controlled or eradicated through phage therapy, and more specifically, the employment of trained phages. First, we employed a single strain of this pathogen originally cultured from a surface wound (Rumbaugh et al. 1999). Recent study shows that *P. aeruginosa* may be highly diverse *in situ*: genetic and phenotypic diversity within individual patients may vary considerably over time (Mowat et al. 2011), and strains sampled may depend on their microenvironments (Goddard et al. 2012). Research suggests that other strains of *P. aeruginosa* can be controlled to some extent by phage, and thus would be amenable to phage training (Morello et al. 2011; Alemayehu et al. 2012), and there is indication that some lytic phages can be cross-infective on different bacterial genotypes or strains (Poullain et al. 2008; Poisot et al. 2011b; Weitz et al. 2013). Second, we did not assess how the passage environment (liquid KB medium) affected phage performance in other (*in vivo*) environments. Study indicates that phage–bacteria coevolution can be influenced by abiotic environments (Lopez Pascua et al. 2012), although it is unclear whether the success of phage therapy is increased as selective and target environments become increasingly similar. Third, although showing a nearly 100% infection capacity, the evolved phages succeeded in reducing bacterial density in liquid culture by only up to one log unit compared with the ancestral phage (Fig. 4). While significant for applications as disinfectants and for surface wounds, this could be insufficient to control or contribute to curing other bacterial infections. It remains to be tested whether phages capable of infecting the vast majority of bacteria in a population could be further improved through serial passage, for example, by increasing their burst sizes.

In conclusion, our results demonstrate the potential for evolutionary insights to improve phage therapy against bacterial pathogens (e.g. Escobar-Páramo et al. 2012; Zhang and Buckling 2012). Phage therapy is a burgeoning area of research (e.g. Pirnay et al. 2012), much of it focussing on safety, industrial production, collection and storage methods (e.g. Hagens and Loessner 2010). However, further study is needed to see how this approach could be put into practice. We need to know how the diversity of target populations (species, strains, genotypes) and environments used for trained phage production (culturing techniques, range of bacterial isolates used, passaged or coevolved trained phage, see Poullain et al. 2008) influence therapeutic outcome parameters, such as host range, the generation of resistant phenotypes and control or eradication of the infection. Depending on needs, phage samples could either be obtained from reference stocks or may be engineered through molecular techniques (e.g. Pouillot et al. 2010) or based on phage training (Morello et al. 2011; Chan and Abedon 2012; Maura et al. 2012). Regarding application of this latter method, an important challenge to trained phage use is the time elapsed between obtaining the bacterial sample for production and actually applying the trained phage to the site(s) of infection. Our experiment evaluated phage after six serial transfers, which would probably be too long a delay for actual therapeutic use. However, time-shift studies on *P. fluorescens* SBW25 indicate that phage gains considerable impact on bacterial populations (evaluated using infection capacity assays) after only a single transfer (e.g. Buckling and Rainey 2002). Finally, it would be interesting to investigate how phage therapy success is affected by (1) producing trained phage on bacteria taken from infected and noninfected areas (Goddard et al. 2012) and (2) the time point(s) at which bacteria are sampled for phage production (Mowat et al. 2011) and at which resultant trained phage are applied.

## Data archiving statement

Data for this study are available in Dryad: doi:10.5061/dryad.5hf57.

## Supporting Information

**Figure S1.** Two photographs illustrating the results of infectivity assays where bacteria are streaked through a line of sample containing purified bacteriophage and viewed under a UV light.

**Figure S2.** Cross-infection matrix used for analysis of specificity of bacterial resistance and of cross-resistance.

**Table S1.** Logistic regression analysis of variation in infection capacity for different phage isolate origins (PEV2, LUZ7, 14/1, LKD16), bacteria type (ancestral t0 vs. evolved t1) and phage type (ancestral t0 vs. passaged t6), in experiment 1.

**Table S2.** Logistic regression analysis of variation in resistance for different bacterial t1 origins (evolved with PEV2, LUZ7, 14/1 or LKD16), tested against t0 assay phage isolates (PEV2, LUZ7, 14/1, LKD16) in the cross-infection assay.

**Table S3.** Analysis of Variance of bacterial cell density (log-transformed), comparing ancestral t0 and evolved t6 phages (phage type) for different phage isolate origins (PEV2, LUZ7, 14/1 or LKD16).
