# Supplementary material for: Back to the future: evolving bacteriophages to increase their effectiveness against the pathogen Pseudomonas aeruginosa PAO1
Source: Evol Appl. 2013 Jul 15;6(7):1054–63. doi: 10.1111/eva.12085 (PMC3804238; doi:10.1111/eva.12085)

**Supplementary Information – Betts, A et al.** Back to the future: evolving bacteriophages to increase their effectiveness against the pathogen *Pseudomonas aeruginosa* PAO1. Evolutionary Applications

**Figure S1**. Two photographs illustrating the results of infectivity assays where bacteria are streaked through a line of sample containing purified bacteriophage and viewed under a UV light. (A) illustrates a population of bacteria completely resistant to the phage applied to the plate, while (B) illustrates a population of bacteria completely susceptible to the phage with complete inhibition of bacterial growth where the phage is encountered.


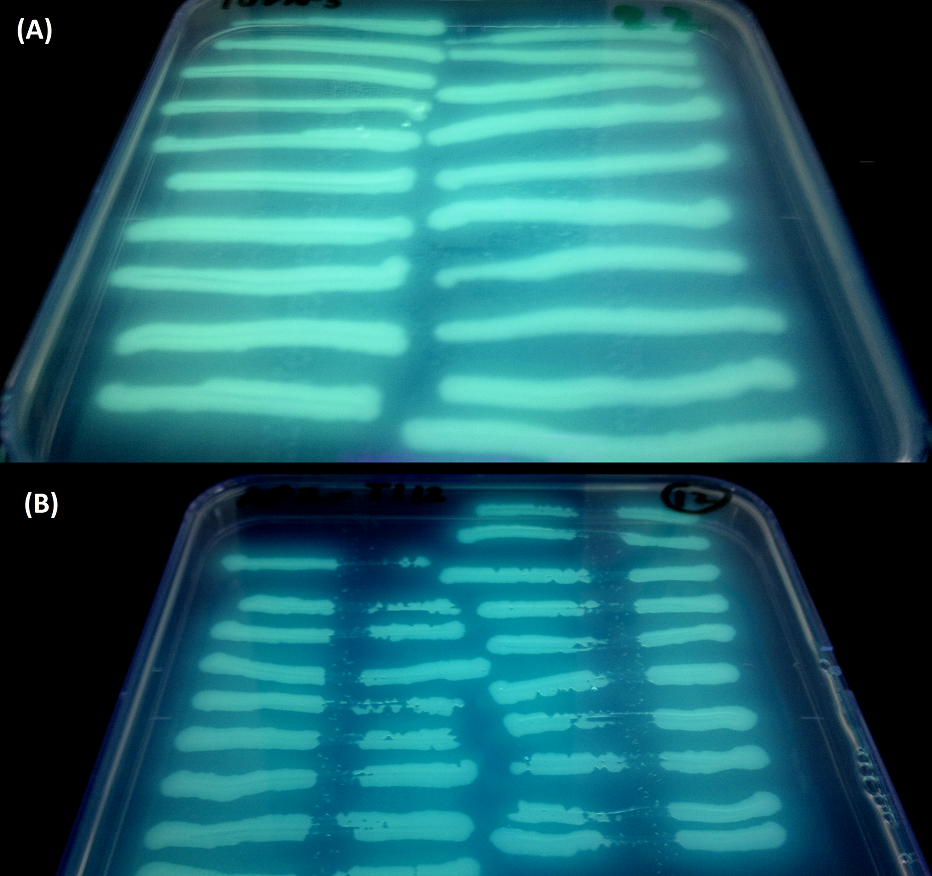

Supplement: Supplementary file 1 [file eva0006-1054-SD1.doc]
