# Supplementary material for: Back to the future: evolving bacteriophages to increase their effectiveness against the pathogen Pseudomonas aeruginosa PAO1
Source: Evol Appl. 2013 Jul 15;6(7):1054–63. doi: 10.1111/eva.12085 (PMC3804238; doi:10.1111/eva.12085)
Supplement: Supplementary file 2 [file eva0006-1054-SD2.doc]

**Supplementary Information – Betts, A et al.** Back to the future: evolving bacteriophages to increase their effectiveness against the pathogen *Pseudomonas aeruginosa* PAO1. Evolutionary Applications

**Figure S2.** Cross-infection matrix used for analysis of specificity of bacterial resistance and of cross-resistance. Bacteria isolated after the first round of serial passage ("t_1_ bacteria"; see Fig. 1) were tested for their resistance to ancestral, unevolved phage ("t_0_ phage"). The diagonal of the matrix represents "sympatric" combinations of bacteria and phage, where bacteria were confronted with their "own" phage isolate, to which they had been exposed for one passage cycle. The off-diagonal represents "allopatric" combinations, with bacteria confronted with "foreign" phage, with which they had no prior contact.
