# Supplementary material for: Back to the future: evolving bacteriophages to increase their effectiveness against the pathogen Pseudomonas aeruginosa PAO1
Source: Evol Appl. 2013 Jul 15;6(7):1054–63. doi: 10.1111/eva.12085 (PMC3804238; doi:10.1111/eva.12085)
Supplement: Supplementary file 3 [file eva0006-1054-SD3.doc]

**Supplementary Information – Betts, A et al.** Back to the future: evolving bacteriophages to increase their effectiveness against the pathogen *Pseudomonas aeruginosa* PAO1. Evolutionary Applications

**Table S1.** Logistic regression analysis of variation in infection capacity for different phage isolate origins (*PEV2*, *LUZ7*, *14/1*, *LKD16*), bacteria type (ancestral t_0_ vs. evolved t_1_) and phage type (ancestral t_0_ vs. passaged t_6_), in experiment 1. To correct for overdispersion, we used a scaled model and calculated mean deviances (2x log-likelihood ratio / d.f.) to perform quasi-F tests. Effects of phage isolate origin, phage type, and their interaction were tested against phage selection line, and the other factors tested against the error term.

| Source | d.f. | Mean Deviance | F |  |
| --- | --- | --- | --- | --- |
| Phage isolate origin | 3 | 243.5 | 361.0 | *** |
| Bacteria type | 1 | 460.0 | 460.0 | *** |
| Phage isolate origin x bacteria type | 3 | 3.1 | 4.5 | * |
| Phage type | 1 | 778.7 | 1154.7 | *** |
| Phage isolate origin x phage type | 3 | 14.9 | 22.1 | *** |
| Bacteria type x phage type | 1 | 0.6 | 0.9 |  |
| Phage selection line[phage type, phage isolate origin] | 28 | 0.7 | 0.7 |  |
| Scaled error | 87 | 1.0 |  |  |

^*^ p = 0.0328; ^***^ p < 0.0001
