# Supplementary material for: Back to the future: evolving bacteriophages to increase their effectiveness against the pathogen Pseudomonas aeruginosa PAO1
Source: Evol Appl. 2013 Jul 15;6(7):1054–63. doi: 10.1111/eva.12085 (PMC3804238; doi:10.1111/eva.12085)
Supplement: Supplementary file 4 [file eva0006-1054-SD4.doc]

**Supplementary Information – Betts, A et al.** Back to the future: evolving bacteriophages to increase their effectiveness against the pathogen *Pseudomonas aeruginosa* PAO1. Evolutionary Applications

**Table S2.** Logistic regression analysis of variation in resistance for different bacterial t_1_ origins (evolved with *PEV2*, *LUZ7*, *14/1* or *LKD16*), tested against t_0_ assay phage isolates (*PEV2*, *LUZ7*, *14/1*, *LKD16*) in the cross-infection assay. To correct for overdispersion, we used a scaled model and calculated mean deviances (2x log-likelihood ratio / d.f.) to perform quasi-F tests. The effect of bacterial origin was tested against bacterial selection line, and the other factors tested against the error term.

| Source | d.f. | Mean Deviance | F |  |
| --- | --- | --- | --- | --- |
| Bacterial origin | 3 | 1.34 | 0.3 |  |
| Assay phage isolate | 3 | 376.9 | 376.9 | *** |
| Bacterial origin x assay phage isolate | 9 | 6.1 | 6.1 | ** |
| Selection line[origin] | 28 | 5.4 | 5.4 | ** |
| Error | 12 | 1.0 |  |  |

^**^ p<0.003; ^***^ p<0.0001
