# Supplementary material for: Back to the future: evolving bacteriophages to increase their effectiveness against the pathogen Pseudomonas aeruginosa PAO1
Source: Evol Appl. 2013 Jul 15;6(7):1054–63. doi: 10.1111/eva.12085 (PMC3804238; doi:10.1111/eva.12085)
Supplement: Supplementary file 5 [file eva0006-1054-SD5.doc]

**Supplementary Information – Betts, A et al.** Back to the future: evolving bacteriophages to increase their effectiveness against the pathogen *Pseudomonas aeruginosa* PAO1. Evolutionary Applications

**Table S3.** Analysis of Variance of bacterial cell density (log-transformed), comparing ancestral t_0_ and evolved t_6_ phages (phage type) for different phage isolate origins (*PEV2*, *LUZ7*, *14/1* or *LKD16*).

| Source | d.f. | Mean Deviance | F | p |
| --- | --- | --- | --- | --- |
| Phage type | 1 | 2.25 | 10.61 | 0.0021 |
| Phage isolate origin | 3 | 5.03 | 7.91 | 0.0002 |
| Phage type x isolate origin | 3 | 2.39 | 3.76 | 0.0166 |
| Error | 48 | 0.21 |  |  |
